# Targeting PI3K/Akt represses Hypoxia inducible factor-1α activation and sensitizes Rhabdomyosarcoma and Ewing’s sarcoma cells for apoptosis

**DOI:** 10.1186/1475-2867-13-36

**Published:** 2013-04-16

**Authors:** Mehtap Kilic-Eren, Tulin Boylu, Vedrana Tabor

**Affiliations:** 1 Department of Medical Biology, Faculty of Medicine, Adnan Menderes University, Aydin, Turkey; 2 Department of Histology and Embryology, Faculty of Medicine, Adnan Menderes University, Aydin, Turkey; 3 Present address: Department of Medical Biochemistry and Biophysics, Karolinska Institute, Stockholm, Sweden

**Keywords:** PI3K/Akt, Hypoxia, HIF-1α, Apoptosis, Rhabdomyosarcoma, Ewing’s sarcoma

## Abstract

**Background:**

Hypoxia inducible factor-1 α (HIF-1α) has been identified as an important novel target in apoptosis resistance of pediatric tumors such as Rhabdomyosarcoma (RMS) and Ewing’s sarcoma (ES). Evidence suggests that PI3K/Akt signaling plays a role in regulation of HIF-1α activation as well as apoptosis resistance in various adult tumors. However the relevance of PI3K/Akt signaling in HIF-1bα activation and apoptosis resistance in childhood tumors has not been addressed yet. Thus, this study was to investigate whether PI3K/Akt signaling is involved in hypoxia induced activation of HIF-1α as well as in resistance to hypoxia-induced apoptosis in childhood tumors such as RMS and ES.

**Methods:**

Constitutive activation of PI3K/Akt signaling was analyzed by Western blotting. Hypoxic activation of HIF-1α was determined by Western Blot analysis and electrophoretic mobility shift assay. Apoptosis was determined by flow cytometric analysis of the propidium iodine stained nuclei of cells treated with PI3K inhibitor LY294002 in combination with either TNF-related apoptosis-inducing ligand (TRAIL) or doxorubicin.

**Results:**

This study demonstrated that PI3K/Akt signaling was constitutively activated in RMS and ES cell lines, A204 and A673, respectively. Targeting PI3K/Akt signaling by the inhibitor LY294002 (30 μM) significantly decreased the protein expression as well as DNA binding activity of HIF-1α and restored the apoptosis-inducing ability of cells in hypoxia Additionally, pretreatment with LY294002 sensitized A204 and A673 cells to TRAIL or doxorubicin induced apoptosis under hypoxia.

**Conclusion:**

These results suggest that the constitutively active PI3K/Akt signaling contributes to hypoxic activation of HIF-1α as well as HIF1α-mediated apoptosis resistance in RMS and ES cells under hypoxia.

## Background

Hypoxia inducible factor-1 (HIF-1) is the major transcription factor activated to mediate adoptive responses under hypoxia [1]. HIF-1 is a heterodimeric protein composed of oxygen regulated α and constitutively active β subunits. When oxygen is present, HIF-1α is hydroxylated by prolylhydroxylases that allows its interaction with von Hippel Lindau (VHL) complex, leading to its ubiquitination and proteosomal degradation. In contrast, when oxygen is not available rate of asparagine and proline hydroxylation decreases and HIF-1α cannot bind to VHL complex and remains stabilized. Stabilized HIF-1α translocates to the nucleus to interact with the co-activators HIF-1β and p300/CBP which results in transcriptional activation of the various genes including growth factors, angiogenic factors, anti-apoptotic factors and the factors involved in anaerobic metabolism [2,3]. HIF-1α is overexpressed in a variety of human tumors associated with poor prognosis and resistance to chemotherapy-induced apoptosis [4]. In our previous work we also identified HIF-1α as an important target modulating apoptosis resistance in pediatric tumors such as Rhabdomyosarcoma (RMS) and Ewing’s sarcoma (ES) [2]. Constitutive activation of phosphatidylinositol 3-kinase (PI3K), due to a variety of genetic aberrations, is frequently observed in human cancers and plays a major role in tumor formation and progression [5,6]. Akt, a serine/threoneine kinase, is a central mediator of the PI3K with several downstream targets. Aberrant activation of PI3K/Akt plays important role in the resistance of tumor cells to anticancer therapy [7,8]. Emerging evidences suggest that PI3K/Akt signaling mediates regulation and activation of HIF-1α in various human cancers [9-11]. However, to date there is no data signifying the relevance of PI3K/Akt signaling in activation of HIF-1α and in resistance to apoptosis under hypoxia in childhood tumors.

RMS is the most common soft tissue sarcoma in children and accounts for 23% of all sarcomas, and 7% of all pediatric malignancies [2,12]. ES is the second most common primary malignant bone tumor [2,13]. Although the majority of RMS and ES patients with non-metastatic disease can be cured, the prognosis of patients with metastatic disease remains inferior [14,15]. Therefore, it is of critical importance to understand the key factors and molecular pathways in pathogenesis and survival of RMS and ES in order to develop novel effective anticancer strategy. Published data indicates that the increased levels of phosphorylated, hence active, Akt in childhood cancer samples, including neuroblastoma, glioblastoma, RMS and ES, is negatively correlated with patient survival [16-20]**.** Accordingly, this study was undertaken to investigate whether constitutive PI3K/Akt signaling is involved in regulation of HIF-1α activation as well as resistance to hypoxia-induced apoptosis in human RMS and ES cell lines A204 and A673, respectively.

## Results

### PI3K/Akt signaling is constitutively activated in A204 RMS and A673 ES cells

**Figure 1:**
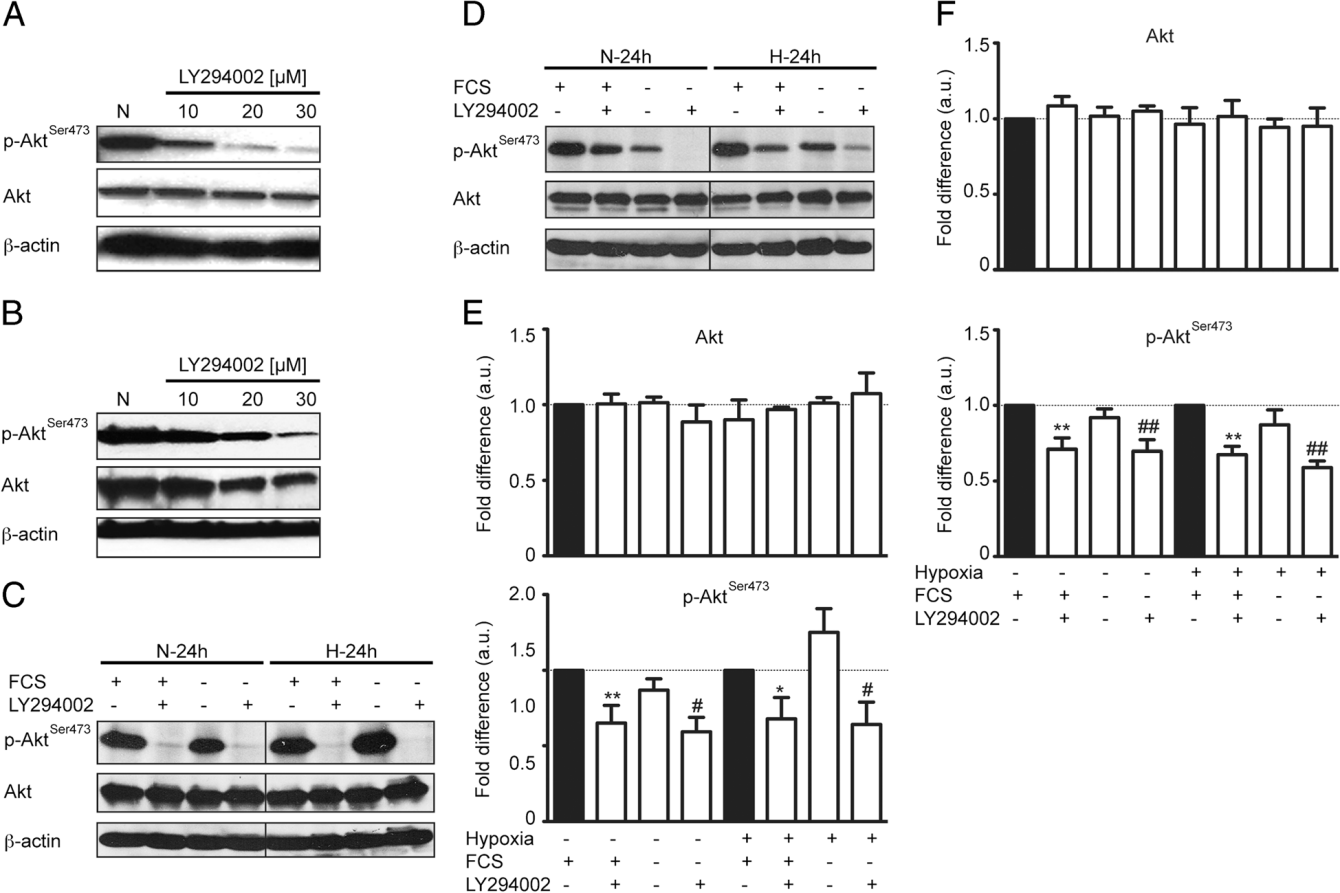
**PI3K/Akt signalling is constitutively activated in A204 and A673 cell lines.** Protein levels and phosphorylation status of Akt and β-actin (loading control), were analyzed by Western blotting in **A**. 24 hours cell culture of A204 cells under normoxia, treated with 0, 10, 20, 30 μmol/L LY294002. **B**. 24 hours cell culture of A673 cells under normoxia, treated with 0, 10, 20, 30 μmol/L LY294002. **C**. 24 h culture of A204 cells under normoxia or hypoxia, medium containing either 0% (−) or 10% FCS (+; *top*) and/or treated with 30 μmol/L LY294002. **D**. 24 h culture of A673 cells under normoxia or hypoxia, medium containing either 0% (−) or 10% FCS (+; *top*) and/or treated with 30 μmol/L LY294002. **E**. Western blots obtained as indicated in C were densitometrically analyzed. Shown are means ± SEM of four independent experiments. Statistically significant differences between LY294002 treated and untreated (20% FCS) are indicated *, p < 0.05 and **, p < 0.01. Statistically significant differences between LY294002 treated and untreated (0% FCS) are indicated #, p < 0.05 and ##, p < 0.01. The difference between untreated hypoxic and normoxic condition was not significant (for 20% FCS, p = 0.1288; for 0% FCS, p = 0.8749). **F**. Western blots obtained as indicated in D were densitometrically analyzed. Shown are means ± SEM of four independent experiments. Statistically significant differences between LY294002 treated and untreated (20% FCS) are indicated *, p < 0.05 and **, p < 0.01. Statistically significant differences between LY294002 treated and untreated (0% FCS) are indicated #, p < 0.05 and ##, p < 0.01. The difference between untreated hypoxic and normoxic condition was not significant (for 20% FCS, p = 0.1529; for 0% FCS, p = 0.3275).

To assess the role of PI3K pathway in HIF-1α induction the phosphorylation status of Akt, which was used as surrogate for PI3K activity, was examined by Western blot analysis. As shown in Figure 1A and B, A204 and A673 cells had high levels of Akt phosphorylation on Ser473 in normoxia. Next we used LY294002, the pharmacologic inhibitor of PI3K to interfere with phosphorylation of Akt, and here we show that upon treatment with LY294002 the level of p-Akt was decreased in a dose dependent manner in both A204 and A673 cells in normoxia (Figure 1A, B). To address whether PI3K/Akt signaling was sustained in hypoxia, phosphorylation of Akt was examined in the presence or absence of LY294002 in both normoxia and hypoxia. In addition, due to the growth factors present in serum, which can induce Akt phosphorylation, we also tested serum-deprived cells. Accordingly, pretreatment of A204 and A673 cells by 30 μM LY294002 decreased phosphorylation of Akt in both conditions whereas protein levels of total Akt were not altered (Figure 1C, D). As seen in Figure 1C and D, levels of p-Akt-Ser473 were similar in A204 and A673 cells either in normoxia or hypoxia and did not change by serum deprivation but suppressed by LY294002 addition. Densitometry analysis also confirmed these data (Figure 1E and F) suggesting in A204 and A673 cells in normoxia p-Akt levels when normalized to Akt levels, is significantly decreased in the presence of LY294002 whether or not FCS is withdrawn. In contrast, no significant differences were detected in p-Akt levels between hypoxia and normoxia in both cells. In hypoxia in A673 cells p-Akt levels again did not change by serum deprivation whereas it seemed to be increased in A204 cells though it was not significant. Moreover, addition of LY294002 was able to suppress p-Akt levels significantly either in the presence or absence of FCS also in hypoxia in both cells. Whereas pretreatment of A204 and A673 cells by 30 μM LY294002 did not alter the protein levels of total Akt. Hence, we conclude that levels of p-Akt were sustained in both cells under hypoxia and did not change by serum deprivation either in normoxia or hypoxia.

Thus, these data demonstrated that activation of PI3K/Akt signaling is constitutive in both cell lines in normoxia and hypoxia, as evidenced by high levels of phosphorylated p-Akt-Ser473, the downstream effector of PI3K (Figure 1C, D).

### PI3-K/Akt signaling is involved in hypoxic induction of HIF-1 alpha protein and DNA binding activity in A204 and A673 cells

**Figure 2:**
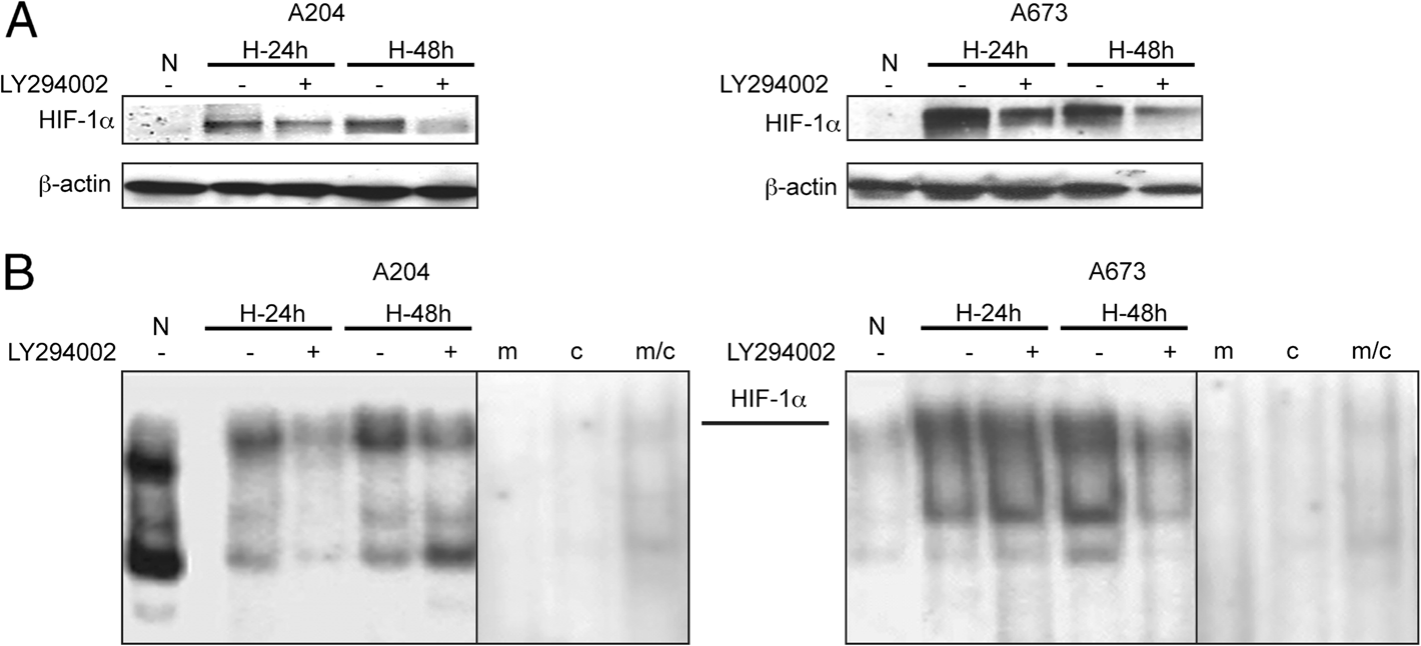
**PI3K/Akt pathway is involved in activation of HIF-1α in A204 and A673 cell lines.** Cells were pretreated with 0 or 30 μmol/L of LY294002 for 1 hour and then incubated for 24 hours or 48 hours either in normoxia (N) or hypoxia (H). **A**. Protein expression levels of HIF-1α and β-actin were analyzed by western blotting. **B**. DNA binding activity of Hif-1α in nuclear extracts was assessed by EMSA. Specific HIF-1 DNA binding was confirmed by using a radioactive labeled mutated (m) probe, in which the HIF-1α consensus binding site is inactivated, and by competition with unlabelled consensus (c) and mutant (c/m) DNA probes in 100 fold excess.

In order to examine whether constitutive activation of PI3K/Akt signaling is involved in hypoxic induction of HIF-1α protein, either pretreated with 30 μM of LY294002, or left untreated (control) A204 and A673 cells were incubated under hypoxic conditions and subsequently subjected to Western blot analysis for stabilization of HIF-1α protein. As shown in Figure 2A, HIF-1α protein was stabilized 24 h after exposure to hypoxia and remained its levels up to 48 h post exposure in both cell lines. Remarkably, pre-treatment with LY294002 decreased the expression of HIF-1α suggesting that induction of HIF-1α by hypoxia requires activation of PI3K pathway. Further, the effect of PI3K inhibitor on hypoxia-induced DNA-binding activity of HIF-1α was investigated by EMSA utilizing a 3^′^ HRE-derived oligonucleotide probe. Using a mutant probe, performing competition assays (Figure 2B) confirmed the identity of the HIF-1α band. Under hypoxic conditions HIF-1α showed increased DNA-binding activity at 24 h and the level of activity was still high at 48 h in both A204 and A673 cells (Figure 2B). Pretreatment with LY294002 reduced hypoxia-induced DNA binding activity of HIF-1α in both cell lines, although this effect was more pronounced in A204 cells after 24 h compared to A673 cells.

### Inhibition of HIF-1α by LY294002 restores apoptosis inducing ability of A204 and A673 cells under hypoxia

**Figure 3:**
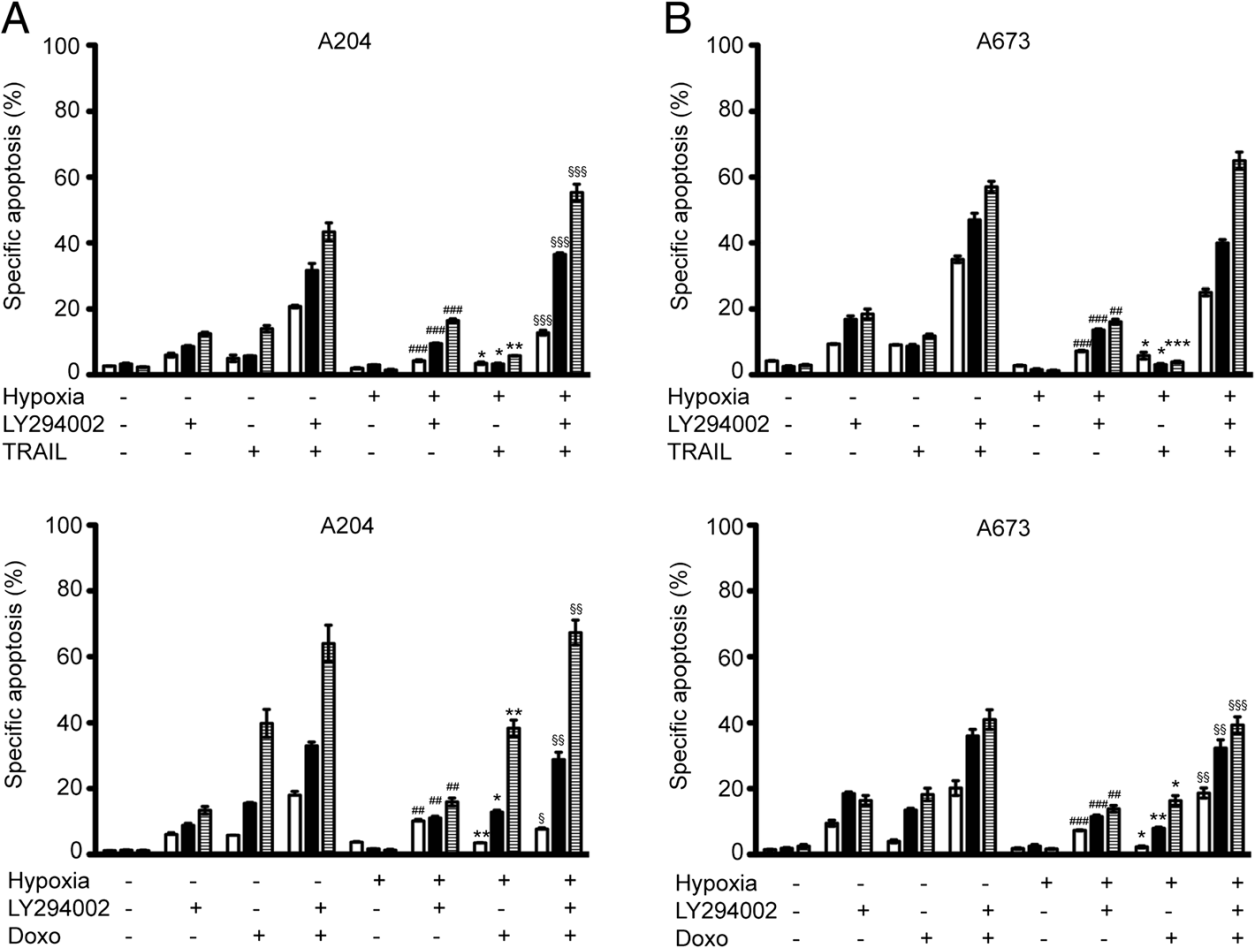
**Sensitization of A204 and A673 cells to doxorubicin- and TRAIL-induced apoptosis by PI3K inhibition.** A204 (**A**) and A673 (**B**) cells were pretreated or not with 30 μmol/L LY294002 for 1 hour, incubated with doxorubicin (0.1 μg/ml) or TRAIL (100 ng/ml) and cultured under normoxic or hypoxic conditions for up to 72 hours. 24 hours-white bars, 48 hours- black bars, 72 hours-hatched bars. Apoptosis was determined by FACS analysis of DNA fragmentation of propidium iodide–stained nuclei; the percentage of specific apoptosis is shown. *Columns,* mean of three independent experiments done in duplicate; *bars,* SD. For statistical analysis two-way *ANOVA* was performed comparing specific apoptosis of either TRAIL or doxorubicin-induced apoptosis under normoxia *vs* hypoxia (*p < 0.05, **p < 0.01, ***p < 0.001) and hypoxia induced apoptosis *vs* hypoxia + LY294002 (^##^p < 0.01, ^###^p < 0.001) as well as hypoxia + TRAIL or doxorubicin vs hypoxia + LY294002 + TRAIL or doxorubicin (^§§^p < 0.01, ^§§§^p < 0.001).

Next, we investigated whether or not decreased stabilization and DNA binding activity of HIF-1α by LY294002, can sensitize A204 and A673 cells to apoptosis under hypoxia. In order to examine long lasting effects of LY294002, cells were cultured for up to 72 h under hypoxic conditions in the presence of 30 μM LY294002 and apoptosis was assessed every 24 hours. As seen in Figure 3A while hypoxia alone did not trigger apoptosis in both cell lines, pretreatment with LY294002 induced 15,5 (±1,7)% and 16,0 (±1,1)% apoptosis in A204 and A673 cells, respectively, in a time dependent manner (Figure 3A). These data suggest that decreased protein level and DNA binding activity of HIF-1α by LY294002 treatment restores apoptosis sensitivity in A204 RMS and A673 ES cells.

In our previous work, we showed that hypoxia protects against death receptor- (*e.g. TRAIL*) and cytotoxic drug- (*e.g. Doxorubicin*) induced apoptosis in A204 and A673 cells. Therefore cells were pretreated with LY294002 and cultured for up to 72 h in the presence or absence of TRAIL in both normoxia and hypoxia. Apoptosis was assessed every 24 hours, and as seen in Figure 3A, without LY294002 pretreatment, after 72 h TRAIL - induced apoptosis in normoxia was at least 10% higher than to that of in hypoxia, underlining the protective role of hypoxia in both cell lines. Interestingly, pretreatment with LY294002 significantly sensitized cells for TRAIL-induced apoptosis and rendered the protective effect of hypoxia (Figure 3A).

Next, the effect of HIF-1α inhibition by LY294002 treatment in combination with doxorubicin, typically triggering apoptosis *via* the mitochondrial pathway, was also tested. In contrast to TRAIL, doxorubicin-induced apoptosis was substantial in A204 and A673 cells under either normoxia or hypoxia, though a slight protective effect of hypoxia was still present. Pretreatment of cells with LY294002 greatly enhanced doxorubicin-induced apoptosis. When pretreated with LY294002 the rate of apoptosis was at least 20% higher in both A204 and A673 cells after 72 h exposure to hypoxia (Figure 3B).

**Figure 4:**
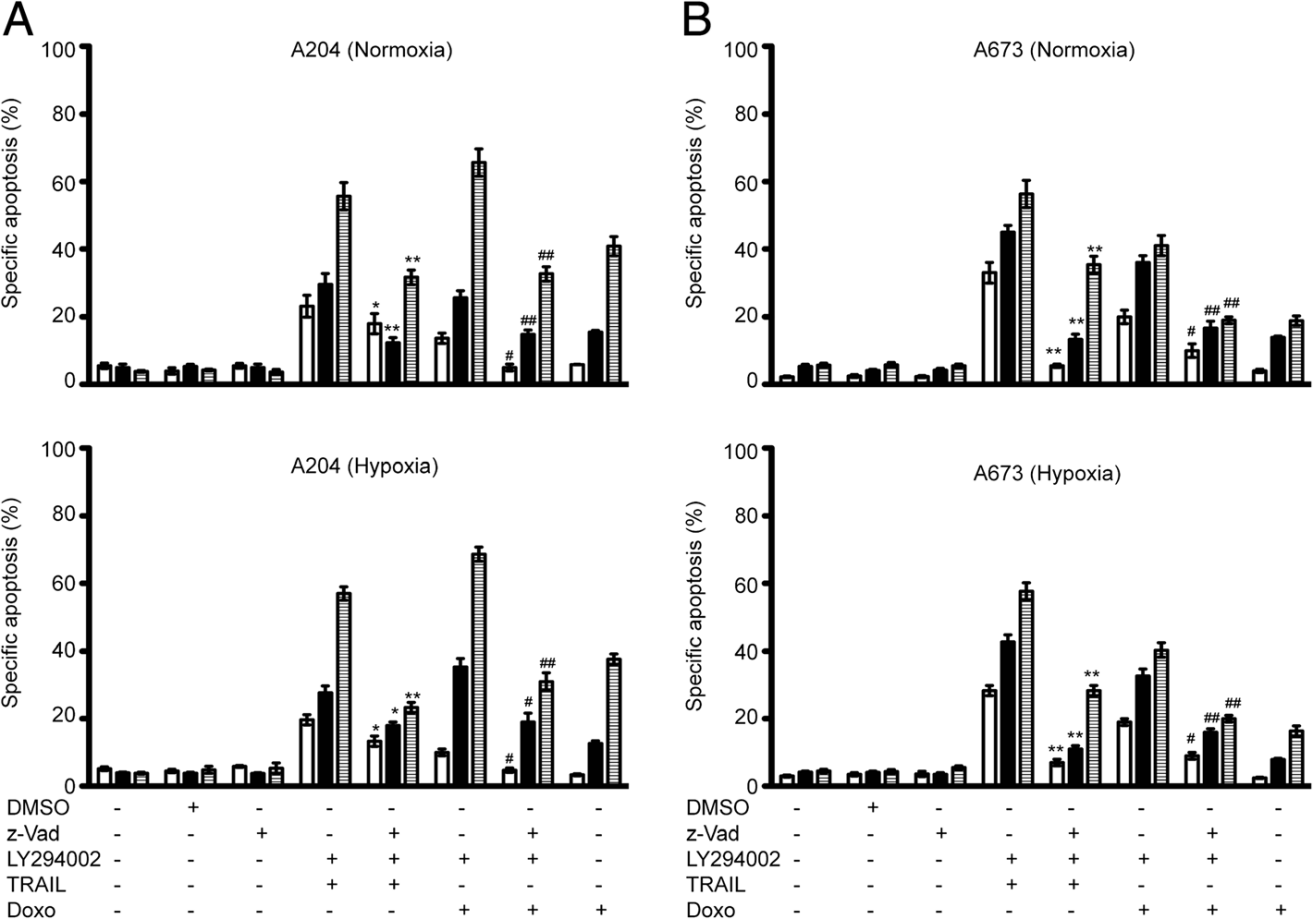
**Sensitization of A204 and A673 cells for doxorubicin- and TRAIL- induced apoptosis is caspase dependent.** A204 (**A**) and A673 (**B**) cells were pretreated with 0 or 30 μmol/L of LY294002 for 1 hour, and cultured under normoxic or hypoxic conditions with doxorubicin (0.5 μg/ml) or TRAIL (100 ng/ml) for up to 72 hours with or without or z-VAD-fmk (50 μmol/L) 24 hours-white bars, 48 hours-black bars, 72 hours-hatched bars *Columns,* mean of three independent experiments done in duplicate; *bars,* SD. For statistical analysis two-way *ANOVA* was performed comparing specific apoptosis of either LY294002 + TRAIL or doxorubicin--induced apoptosis without z-VAD-fmk v*s* with z-VAD-fmk under normoxia (*p < 0.05, **p < 0.01) or the same comparison of specific apoptosis under hypoxia (^#^p < 0.05, ^##^p < 0.01).

Moreover, the broad range caspase inhibitor z-VAD-fmk was used to test requirement for caspases during TRAIL- or doxorubicin-induced apoptosis under hypoxia. Apoptosis induced by combined treatments of LY294002 and TRAIL, or doxorubicin was significantly blocked in the presence of z-VAD-fmk under both normoxia and hypoxia in both cell lines in a time dependent manner (Figure 4A and B). These results indicated that apoptosis induced by combined treatments with LY294002 and either TRAIL, or doxorubicin was mediated by caspases.

## Discussion

Previously, HIF-1α has been identified as key factor in conferring resistance to apoptosis under hypoxia in childhood tumors such as RMS and ES [2]. Evidences suggest that PI3K/Akt signaling plays a role in regulation of HIF-1α activation in various adult tumors [8,21-25]. The present study was undertaken to investigate the relevance for PI3K/Akt signaling and HIF-1α activation along with apoptosis resistance in RMS and ES. Here, it is presented for the first time that constitutively activated PI3K/Akt involved in hypoxic activation of HIF-1α and targeting PI3K/Akt via LY294002 prevented HIF-1α’s stabilization and restored apoptosis sensitivity of RMS and ES cells under hypoxic conditions.

The current study provides a number of evidences supporting this conclusion. First, we demonstrated that A204 (RMS) and A673 (ES) cell lines show high level of phosphorylated Akt (on Ser473), sustaining under serum deprivation and hypoxia indicating to the constitutive activation of Akt. Our data is consistent with the in vivo studies, showing the increased abundance of active, phosphorylated, Akt in several childhood cancers, including RMS and ES [16-20,26]. Second, Akt phosphorylation was inhibited *via* PI3K inhibitor LY294002 that also decreased the protein expression and DNA binding activity of HIF-1α. More importantly inhibition of PI3K/Akt signaling or HIF-1α activity by LY294002 blocked protection against hypoxia-induced cell apoptosis. Third, inhibition of HIF-1α activation via LY294002 also sensitized RMS and ES cells for death receptor (TRAIL) - as well as drug (Doxorubicin) - induced apoptosis which could be blocked in the presence of z.VAD. fmk.

Oxygen regulated transcription factor HIF-1α and the serine/threonine kinase Akt are both essential for development and implicated in tumor growth [8,27-31]. They share the ability to induce processes such as angiogenesis, glucose uptake, and glycolysis [29,32-34]. To date several studies have identified the PI3K/AKT pathway as an important element in hypoxic induction of HIF-1α protein and activity in tumor cell lines [9,11,35,36] Also, in non malignant systems such as developing rat brain or pulmonary artery smooth muscle cells PI3K/Akt pathway is involved in activation of HIF-1α [21,37,38].

From our data, we propose that constitutive activation of the PI3K/Akt contributes to the increased hypoxic activation of HIF-1α in RMS and ES cells, because inhibiting PI3K/Akt activity by the inhibitor LY294002 decreased HIF-1α protein levels and prevented DNA binding activity under hypoxia. However, there are other reports indicating the contrary data and suggesting that PI3K/Akt signaling is neither required nor sufficient for the hypoxic stabilization or activation of HIF-1α [39,40]. Hence, one possibility is that the involvement of constitutive PI3K/Akt signaling in hypoxic activation of HIF-1α may depend on cell type or on tumor type/stage and its microenvironment.

The PI3K/Akt pathway is also well known to mediate prosurvival signals. In particular, Akt is involved in inhibition of apoptosis by phosphorylating pro-apoptotic molecules *i.e. Bad, Caspase-9* or modulating transcription factors *i.e. c-Raf.*[41]. Recent studies have shown that inhibition of PI3K/Akt might be a promising strategy to decrease the threshold for apoptosis induction via the death receptor triggering or cytotoxic drugs in neuroblastoma and glioblastoma [8,26,42]. In line with that our data also provides evidence that PI3K/Akt inhibition cooperates with TRAIL or doxorubicin to trigger apoptosis under hypoxia in RMS or ES cells. Resistance to apoptosis is still major obstacle in treatment and our findings may have important implication for apoptosis-based therapy of RMS and ES. Moreover it provides basis for further investigation of new generation PI3K inhibitors in combination with TRAIL or chemotherapy to overcome apoptosis resistance associated with tumor hypoxia. Similarly a previous report also suggests 3-phosphoinositide-dependant kinase-1(PDK-1)/Akt pathway as an attractive therapeutic target in RMS [17].

It will be the object of our further investigations to elucidate the exact role of PI3K/Akt in hypoxic activation of HIF-1α and to identify the molecules mediating the sensitization effect of PI3K/Akt.

## Conclusion

Constitutive activation of PI3K/Akt involved in hypoxic activation of HIF-1α in RMS and ES cells. Targeting PI3K/Akt via LY294002 prevented HIF-1α’s stabilization and restored apoptosis sensitivity of RMS and ES cells under hypoxic conditions. The current study identifies an important link between PI3K/AKT and HIF-1α, which may have particular relevance to disease progression as well as therapeutic target for cancer intervention in RMS and ES.

## Materıals and methods

### Cell Culture and Hypoxia incubation

Human Rhabdomyosarcoma (A204) and Ewing’s sarcoma (A673) cell lines were obtained from American Type Culture Collection (Manassas, VA) and were grown in Dulbecco’s modified Eagle’s medium (Life Technologies, Inc., Eggenstein, Germany) containing 10% heat inactivated fetal calf serum (Biochrom, Berlin, Germany), 100 IU/ml penicillin, 100 μg/ml streptomycin (Biochrom), 10 mM glutamine (Biochrom) in a humidified atmosphere at 37 °C with 5% CO_2_ unless otherwise specified. Hypoxic conditions (0.5% O_2_) were achieved by incubation in a humidified internal incubator of a hypoxia glove box (Coy Laboratory Products, Inc.). After an initial exposure to low oxygen, all subsequent treatments were given within the glove box to prevent cellular damage due to reoxygenation.

### Determination of apoptosis

Apoptosis was assessed by fluorescence-activated cell-sorting (FACScan, Becton Dickinson, Heidelberg, Germany) analysis of DNA fragmentation of propidium-iodide stained nuclei as described previously [2]. The percentage of specific apoptosis was calculated as follows: 100 × [experimental apoptosis (%) − spontaneous apoptosis (%)] [100% − spontaneous apoptosis (%)].

### Protein extraction and Western blot analysis

Total cell extracts were prepared from cells grown in 6-well plates at 90% confluence. Cells were exposed to 20% O_2_ or 0.5% O_2_ for the indicated time points and lysed in lysis buffer (20 mM Tris, pH 7.5 (Sigma), 150 mM KCl (Sigma), 1 mM EDTA, 1% Triton X-100 (Sigma) supplemented with protease inhibitor mixture (Complete®; Roche Applied Science, Mannheim, Germany). 0.2 mM phenylmethylsulfonyl fluoride (PMSF); 0.5 mM dithiothreitol (DTT) and 1 mM sodium-ortho-vanadate before use. Western blot analysis was done as described previously using primary antibodies, mouse anti-Hif-1α monoclonal (1:250; BD Biosciences; Heidelberg, Germany), rabbit anti-phospho Akt (Ser 473) (D9E) and rabbit anti-Akt (Cell Signaling, Beverly, MA), followed by goat-anti-mouse IgG or goat-anti-rabbit IgG conjugated to horseradish peroxidase (1:5,000; Santa Cruz Biotechnology) [2]. Mouse anti-β-actin monoclonal antibody (1: 5000; Sigma), was used as a loading control. All proteins were visualized using enhanced chemiluminescence (Amersham Biosciences).

### Nuclear protein extraction and Electrophoretic mobility shift assay (EMSA)

Nuclear extracts were prepared essentially as described [2]. Oligonucleotide DNA probes containing the HIF-1α binding sequence 5^′^-TCTGTACGTGACCACACTCACCTC [43] and a mutant probe 5^′^-TCTGTAAAAGACCACACTCACCTC, labelled with with γ-32P-ATP (Amersham, Freiburg, Germany) and annealed with complementary oligonucleotides, were used for EMSA [43]. EMSA was done as described previously [2]. In general, unless otherwise stated, all qualitative analyses were repeated at least three times.

## Abbreviations

PI3K: Phosphatidylinositol 3-kinase; Akt: Protein kinase B (PKB); HIF1α: Hypoxia inducible factor 1 alpha; HIF1β: Hypoxia inducible factor 1 beta; RMS: Rhabdomyosarcoma; ES: Ewing’s sarcoma; TRAIL: TNF-related apoptosis-inducing ligand; VHL: Von Hippel Lindau; CBP: CREB binding protein; EMSA: Electrophoretic mobility shift assay; PDK: 1-3-phosphoinositide-dependant kinase-1.

## Competing interests

“The authors have declared that no competing interest exists”.

## Authors’ contribution

MKE was involved in the planning and supervision of the project, funding acquisition from received grant, conducting the experiments, data analyses and interpretation, writing and revision of the manuscript. TB and VT were involved in conducting experiments, data analyses and interpretation and drafted the article. All authors have contributed and approved the final manuscript.
